# Evaluating the Drinking Water Distribution System of Lahore for Free-Living Amoebas, Particularly *Naegleria* spp.

**DOI:** 10.1155/japr/4286995

**Published:** 2025-07-26

**Authors:** Ayesha Najam, Sana Ullah Iqbal, Waqas Ahmed, Rabia Tanvir, Haroon Akbar

**Affiliations:** ^1^District Food Laboratory, Punjab Food Authority, Lahore, Pakistan; ^2^Department of Food Science and Human Nutrition, University of Veterinary and Animal Sciences (UVAS), Lahore, Pakistan; ^3^Institute of Microbiology, University of Veterinary and Animal Sciences (UVAS), Lahore, Pakistan; ^4^Department of Parasitology, University of Veterinary and Animal Sciences (UVAS), Lahore, Pakistan

**Keywords:** amoeba, freshwater, *Naegleria*, primary amoebic meningoencephalitis

## Abstract

Free-living amoebas are ubiquitous in distribution systems and recreational waters. Numerous studies have described the problem posed by their presence in the drinking water distribution systems of Lahore; however, very few studies have been done on *Naegleria* spp., particularly *Naegleria fowleri* that causes primary amoebic meningoencephalitis. In this study, we aimed to screen for the free-living amoebas in 100 water samples from nine zones in Lahore. These samples included water from mosques (*n* = 45), homes (*n* = 45), swimming pools (*n* = 10), and the Lahore canal. Cysts (3–4 *μ*m) and rounded trophozoites (4–5 *μ*m) along with elongated trophozoites (8–10 *μ*m) were observed to be present in 37 (82.2%) water samples from mosques. In water from homes, we detected rounded trophozoites (5 *μ*m) and elongated trophozoites (10 *μ*m) from the Lahore canal. There was also a positive association with temperature (odds ratio 20.329, 95% CL) and a trend of negative association with pH (odds ratio 2.001, 95% CL). PCR amplification confirmed the presence of *Naegleria* spp. in three zones: Lahore canal and swimming pools. Our study indicates the presence of *Naegleria* spp. in drinking water distribution systems of Lahore; therefore, we recommend a routine screening for *N. fowleri* in order to reduce the risk of acquiring the fatal primary amoebic meningoencephalitis infection.

## 1. Introduction

Free-living amoebas (FLAs) are the group of protists, which are distributed across soil and freshwater environments [1]. Originally found in the terrestrial biomes, they have been studied for their presence in drinking water distribution systems (DWDSs) and in recreational waters such as swimming pools [1, 2]. Among them is a genus *Naegleria* that is amphizoic and widely distributed as both free living and parasites. These species include *Naegleria fowleri*, *Naegleria italica*, *Naegleria philippinensis*, and *Naegleria australiensis* [3]. Among them, *N. fowleri* is the only one recognized as pathogenic. It is commonly known as “brain-eating amoeba” and is the causative agent of primary amoebic meningoencephalitis (PAM), a rare but fatal infection with a mortality rate of 95%–99%. Recent studies have shown that Pakistan has the second highest prevalence of *Naegleria* infections globally, with reported cases surpassing those documented in the United States over the past 50 years [4]. Since *N. fowleri* is also a thermophile, its infections are increasing with the increasing heat waves due to climate change [5]. Pakistan is among those countries that have been vulnerable to them because of its geographical location displaying the devastating effects of climate change. Despite producing only < 1% of the world's greenhouse gases, the effects of climate change have been disastrous especially in the form of worsening heat waves. Such temperatures have resulted in the emergence of *N. fowleri* as a growing problem of the water distribution system in the country [4]. Studies have reported that the majority of the cases are registered during the summer and premonsoon period. Such studies have focused on understanding the correlation of climate change with the presence of *N. fowleri* in the water bodies of Pakistan [6].

Although *N. fowleri* normally resides in freshwater habitats with temperatures above 35°C up to 46°C for its growth such as rivers, lakes, and even in hot water springs, in Pakistan, it has been reported in reservoirs that contain nonchlorinated water from the municipal water supply [5, 7]. In the megacity of Lahore, water scarcity is common; a reliance on water storage tanks has increased the risk of acquiring its infection [8]. Being the second most populated city in Pakistan and also being a provincial capital, the need for storing water in Lahore is even greater because of the decreasing level of groundwater. A point to consider is that all the water used for drinking in the city is acquired by drilling and storage is done in water tanks prior to chlorination or filtration [2]. In the summertime (April to June), temperatures normally soar higher than 40°C reaching 48°C–49°C in the recent years due to the unfortunate impact of climate change. *N. fowleri* is reported to thrive in hot temperatures (40°C–46°C) with its associated infections mostly reported throughout April to September since the summer has become longer—another impact of climate change. Not to mention, the humid conditions prevailing in monsoon (June to September) and poor chlorination of the domestic water supplies make the conditions even more favorable for its proliferation [9, 10].

FLA lifecycle involves a vegetative form (trophozoite) and a nonvegetative dormant form (cyst); however, *Naegleria* spp. also possess an additional form (flagellate) [1]. Previous studies have reported that *N. fowleri* can infect from the olfactory route from where it enters the nasal cavity and travels to the brain. There it results in a lethal infection called PAM [11], with a mortality rate of 95%–97% [7, 12]. The chances of acquiring this infection are higher in individuals performing activities that render water in the nasal cavity. One example is a ritual in Islamic faith called ablution, that is, rinsing of the nasal cavity with water. Since Pakistan is a Muslim country, this practice is common before offering of prayers in a mosque [7, 13]. Previous studies have speculated that the patients for PAM infection could have acquired the amoeba through this route [14] by using municipal water [1].

The incidence of diseases associated with FLA-related diseases has been increasing in DWDS [1]. For our study, the foundational data and contextual background were provided by the first report on the occurrence and distribution of pathogenic FLAs in the DWDS of Pakistan, conducted by Yousuf et al. [8]. This study highlighted the presence of *Acanthamoeba* spp. and *N. fowleri* in 38% of DWDS samples in Karachi, with 8% of the samples containing *N. fowleri*. Their findings prompted the need for continued surveillance, and the screening of FLAs from water samples stored in domestic tanks in the study directed our approach. Given the similar water storage practices in Lahore, we extended our assessment to detect the presence of FLAs, particularly *Naegleria* spp., in its water distribution system.

## 2. Material and Method

### 2.1. Sampling and FLA Staining

**Figure 1:**
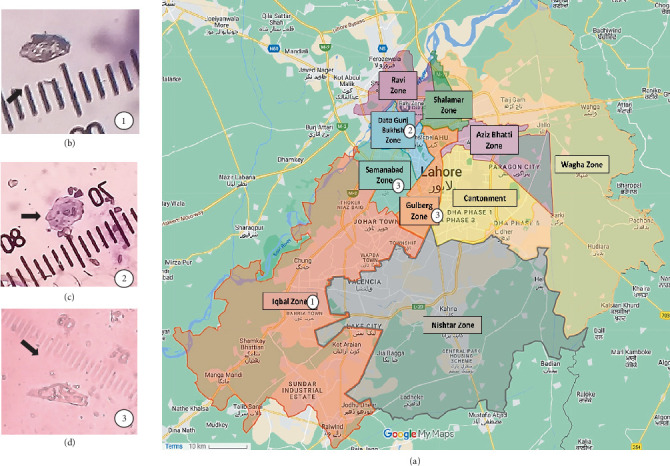
(a) Map depicting the location of the nine zones of the Lahore city according to the City District Government Lahore (CDGL) (source: Google My Maps). The numbers relate to the three trophozoites confirmed to be of free-living amoebas (FLAs) in the following zones: (1) Iqbal zone (pink) (2) Data Gunj Bakhsh zone (green), (3) Samanabad zone (blue), Gulberg zone (orange). (b) Rounded trophozoites (5 *μ*m). (c) Rounded trophozoites (5 *μ*m). (d) Elongated trophozoites (10 *μ*m); stage micrometer 100 mm.

In order to determine the presence of FLAs particularly *Naegleria* spp. in water samples of Lahore, Pakistan (31°32⁣′58.99⁣^″^N, 74°20⁣′37⁣^″^E), a total of 100 samples were collected from nine zones (Ravi zone, Shalamar zone, Wagha zone, Aziz Bhatti zone, Gulberg zone, Samanabad zone, Data Gunj Bakhsh zone, Iqbal zone, and Nishtar zone) across the city (Figure 1). The reason for choosing these locations was that the City District Government Lahore (CDGL) has divided the city of Lahore into these nine zones [15]. The samples included water from mosques (45/100), homes (45/100), and recreational places (10/100) including the Lahore canal and private swimming pools in the area. The samples were collected from April to September with average temperatures ranging from 25°C to 45°C. They were taken midstream in 2-L narrow-mouth sterile bottles and kept at 4°C until processing within 24–48 h [16]. pH and temperature were taken at the site at the time of sampling.

The collected sample was centrifuged at 4000 rpm for 5 min to obtain a sediment pellet. Giemsa staining was performed on this pellet as described by Hebbar et al., [17], and the slides were observed under a microscope at 10X and 40X magnifications. Only the samples showing cysts and trophozoites were further processed for culturing.

### 2.2. FLA Culturing

The sediment pellets were suspended into two media, 1 mL of Page's amoeba saline media [18] poured into nonnutrient agar with a lawn of *Escherichia coli* [19] and nutrient agar with heat-killed *E. coli* [20]. The plates were incubated at room temperature and checked daily for plaque formation. The number and size of the plaques were documented.

### 2.3. DNA Extraction and PCR

DNA was extracted using the method of Moré et al. [21] with little modification. Briefly, the amoeboid plaques were cut from the media plates into small pieces to add to a PowerBead tube. Further protocol was followed as provided by the Exgene Soil DNA mini kit (GeneAll Biotechnology, Korea) manufacturer. The DNA purity and yield were determined using ND-1000 NanoDrop spectrophotometer (Thermo Scientific, United States).

The PCR amplification was carried out for *Naegleria* spp. using the primers Fw2 and RV2 as mentioned by Panda et al. [22]. Before the amplification, a gradient PCR was applied to determine the annealing temperature of the primer sets as described by Porta and Enners [23]. A temperature range of 51°C–55°C was set according to the melting temperatures (*T*_m_) of the primers (*T*_m_–5°C) [24]. After determination of the annealing temperature, the PCR amplification reaction was carried out with a final volume of 25 *μ*L of reaction mixture containing 75 ng of DNA, and the PCR was carried out in 30 cycles with initial denaturation (94°C, 30 s), annealing (55°C, 30 s), and extension (72°C, 30 s). The amplified products were visualized by agarose gel electrophoresis using a 2% gel. The expected product for *Naegleria* spp. was between 408 and 457-bps fragment [22].

### 2.4. Statistical Analysis

Analysis was done using partial coefficient analysis with three variables, temperature, pH, and the presence of trophozoites. Ordinal regression model using logit link function was also run on the data to determine the association with temperature and pH with the predictor variable being the presence of trophozoites. The analysis was carried out using SPSS software Version 29.0 (SPSS, https://www.ibm.com). A value of *p* ≤ 0.05 was considered as statistically significant.

## 3. Results

### 3.1. Giemsa Staining and FLA Culturing for Amoeboid Plaque Formation

The staining of the pellet revealed that at 10X and 40X magnification, out of the 45 samples for mosque water, 37 (82.2%) samples contained cysts and trophozoites. The cysts (3–4 *μ*m) and rounded trophozoites (4–5 *μ*m) were observed along with elongated trophozoites (8–10 *μ*m) which also displayed amoeboid plaques (Figure 1). In a sample from Shalamar zone's Baghbanpura mosque (2.2%), no plaques were observed, and in a sample from Gulberg zone's Fazil mosque (2.2%), no trophozoites were seen; however, amoeboid plaques were observed. Majority of the samples, that is, 16, showed only 1 plaque formation, 8 water samples showed 0 plaque formation, and 1 sample showed 11 plaque formation (Table 1).

In the case of water from domestic taps, we detected rounded trophozoites (5 *μ*m) and plaque formation in 35 (77.7%) samples, and no trophozoites and/or plaques were seen in 10 (22.2%) samples. The highest number of samples (12) showed only 1 plaque formation and 11 samples resulted in 0 plaque formation whereas 1 sample showed as many as 10 plaque formations. The water from the famous Lahore canal contained elongated trophozoites (10 *μ*m) (Figure 1) that resulted in plaque formation as well. Although trophozoites were observed in one water sample of a private swimming pool, no plaques were observed in this or the other two samples (*n* = 3, 0%).

### 3.2. Effect of Temperature and pH

The water samples were taken during the duration of the entire summer season, that is, April till September; therefore, the average temperature ranged from 25°C to 45°C. Among the water samples, the highest temperature was recorded to be 45°C from a Begum Kot home and 44°C from a Badami Bagh home. Both homes were located in the Ravi zone area. In four other samples, one from a home in the Shalamar zone, the temperature was recorded as 43°C, and in three samples from mosque waters (Data Gunj Bakhsh, Samanabad, and Ravi zones), the temperature was recorded as 42°C. The presence of trophozoites was also noted for these samples, and ordinal regression model using logit link function was run on the data. It was observed that an increase in temperature (≥ 38°C) was associated with an increase in the odds of the presence of trophozoites with an odds ratio of 20.329 (95% CL).

**Figure 2:**
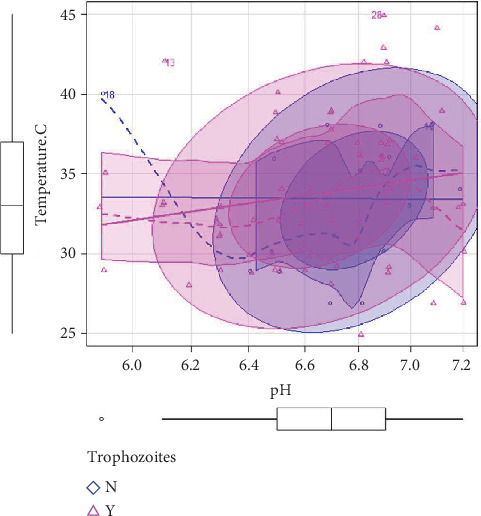
A bivariate scatterplot diagram with shaded ellipses displaying the association pattern of the presence and absence of free-living amoebas (FLAs) trophozoites with temperature and pH of water. Pink triangles (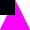) represent positive samples, while blue circles (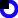) represent negative samples. The shaded ellipses indicate the 95% confidence regions, showing the typical range of pH and temperature values for each group. Solid lines represent the overall trend, and dashed lines represent smoothed patterns in the data. Boxplots along the axes show the distribution of pH and temperature values across all samples.

The pH values were also determined for the samples, and a variation was noted with the pH ranging from 5.9 to 7.2. Using the ordinal regression model with a logit link function, it was determined that an increase in pH (7.0) was associated with a decrease in the presence of trophozoites with an odds ratio of 2.001 (95% CL) (Table 1). A bivariate scatterplot diagram with shaded ellipses corresponding to 95% confidence intervals for each group also displayed a similar trend for pH and temperature (Figure 2). It supported the suggestion that trophozoites were primarily detected in water samples with moderately high temperatures (30°C–38°C) and near-neutral pH levels (6.4–7.0). Despite an overlap in the temperature and pH between positive and negative samples, slight differences in their central trends suggest preferential growth conditions for the trophozoites.

### 3.3. Detection of *Naegleria* spp.

**Figure 3:**
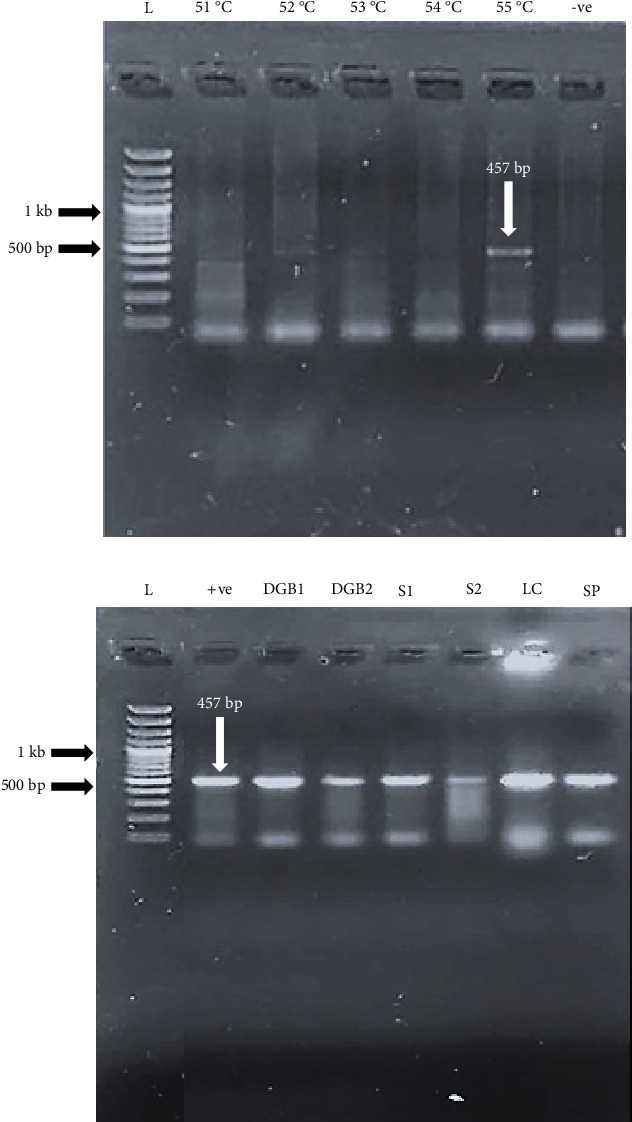
(a) Gradient PCR for the establishment of the annealing temperature for the primers Fw2 and RV2 for *Naegleria* spp. An amplicon size of 457 bp indicated a positive result. Left to right: Lane 1: 100-bp DNA ladder, Lanes 2–5: 51°C–55°C annealing temperature, Lane 6: −ve control. (b) Confirmation of *Naegleria* spp. through conventional PCR. An amplicon size of 457 bp indicates a positive result. Left to right: Lane 1: 100-bp DNA ladder, Lane 2: +ve control, Lane 3: Data Gunj Bakhsh zone tap water (DGB1), Lane 4: Data Gunj Bakhsh zone mosque water (DGB2), Lane 5: Samanabad zone tap water (S1), Lane 6: Samanabad zone mosque water (S2), Lane 7: Lahore canal (LC), Lane 8: private swimming pool (SP).

The gradient PCR exhibited a light band for the 408-bp amplicon for *Naegleria* spp. at the annealing temperature of 52°C; a light band for the 457-bp amplicon for the *Naegleria* spp. was observed at the temperatures 52°C and 53°C. A bright band for the 457-bp amplicon was seen at the temperature 55°C (Figure 3a).

For the PCR amplification, the amoeboid plaques of samples from the same sources were pooled together. The positive amplicon of 457 bp was observed for the *Naegleria* spp. in the water samples of mosques and tap water from Data Gunj Bakhsh zone and Samanabad zone. This amplicon was also seen in the pooled tap water samples of Iqbal zone; however, no amplicons were observed in its mosque water. The pooled samples for mosques and tap water from the remaining six zones showed no amplification of the 457-bp amplicon. The samples from the Lahore canal and the pooled samples from the private swimming pools also showed the positive amplicon for *Naegleria* spp. (Figure 3b).

## 4. Discussion

The presence of FLA has become an increasingly important issue in the DWDS and recreational water facilities. It is because they are ubiquitous organisms and are able to thrive in a variety of environmental conditions [5]. Pakistan has the second highest prevalence rate of infections caused by *Naegleria*. In the previous year, it caused the loss of seven lives in different cities, with one death reported from Lahore [6]. Apart from a few recent studies on the presence of FLA in the waters of Lahore, very little information exists, especially on the presence of amoeba that causes serious infections such as PAM in this megacity. Therefore, to the best of our knowledge, our study is the first of its kind that involves the screening of DWDS for FLAs provided to homes, mosques, and recreational waters in the nine zones of Lahore. A recent study by Nadeem et al. [4] corroborates this fact that no study has researched the cases of *N. fowleri* from Lahore in the past 8 years, and the data on its infections are either not considered or missing from the health care settings.

Our study showed that cysts and trophozoites were present in the water from both mosques and homes along with the water from the famous Lahore canal. It is not surprising since the canal water is muddy and used for irrigation purposes. However, what is alarming is that this canal is a famous spot for swimming by the locals and at one time as many as thousands of people were seen swimming in it to cool off from the scorching heat [25]. More alarming is the fact that these cysts and trophozoites were observed in the DWDS supplied to the mosques and homes through the water and sanitation agency (WASA), Lahore (https://wasa.punjab.gov.pk). They claim that they carry out the treatment processes through chlorination and sometimes filtration. However, as mentioned by a previous study, there is no monitoring implemented for its proper functioning [4] which is indicative of the results of our study. It should be mentioned that the presence of biofilms in the water distribution system might also contribute to the presence of the cysts and trophozoites. The pipe wall biofilms have been found to promote and proliferate the growth of amoeba and protect them against the commonly used disinfectants such as chlorine. Other factors that may be contributing to the presence of these FLAs may include distance from the treatment plant, less concentration of residual chlorine, material of the pipes, and higher water temperature [1]. The factor related to high water temperature is especially notable in our study where the temperature of the water samples ranged from 25°C to 43°C in the duration of the summer months (April to September). It is a possibility that the increased number of cysts and trophozoites observed in our study may be due to this factor. This is also supported by the positive result of PCR amplification for *Naegleria* spp. in the water from both mosques and domestic taps from different zones of Lahore. Therefore, it can be said that there was a trend of positive association with temperature for the presence of trophozoites.

Interestingly, however, a trend of negative association was observed with pH despite previous studies reporting the effect of pH to be negligible [26, 27]. It may be because these studies mention this effect in untreated water (brackish water or groundwater aquifers) and not in treated water as in our study. Our findings support the established fact that the alkaline pH might support and enhance the nonviability of *Naegleria* spp. [28]. This conclusion falls in line with the known treatment of recreational and drinking water reservoirs for enhancing human safety through chlorination. Since liquid chlorine is alkaline, it can raise the pH for better disinfection against *Naegleria* spp. Still, there may be a problem if excessive chlorination is carried out as pointed out by Kumar et al. [29] that it results in disinfection byproducts such as trihalomethanes (THMs) that are carcinogenic. Therefore, a carefully monitored and expert use of chlorination is recommended against them. A study by Zahid et al. [2] on the effect of chlorine in drinking water in Lahore described that a concentration of 5 mg/L^−1^ was required to stop the growth of trophozoites and cysts of *Allovahlkampfia spelaea*, *Vermamoeba vermiformis*, and *Acanthamoeba* spp. Among these protozoans, *Allovahlkampfia spelaea* has been reported previously to belong to the Vahlkampfiidae family that are *Naegleria-*like in morphology, frequency of isolation, environment, and thermotolerance [30]. Therefore, it can be assumed that the concentration described for *Allovahlkampfia spelaea* can be applied for the *Naegleria* spp., though more studies are required to test this assumption.

The samples in our study tested positive for *Naegleria* spp., which may include the species responsible for PAM. A previous study by Nageeb et al. [30] confirmed that out of the 47 *Naegleria* species, the only species that caused PAM was *N*. *fowleri*. Therefore, the possibility that *Naegleria* spp. were found in the water samples is alarming because the chances of the presence of *N. fowleri* are there. The cases of PAM reported in Lahore also suggest this possibility as one case was reported in 2017 [31] and the latest was reported in 2023 [4]. In the water samples of two zones (Data Gunj Bakhsh zone and Samanabad zone) of the city, *Naegleria* spp. were indicated in the municipal water provided to mosques and taps, and it was alarming to note that both the zones were of the oldest and the most densely populated part of the city. Not to mention the DWDS in these areas needs continuous monitoring, and a study by Fatima et al. [32] showed that the water from Samanabad zone contained coliforms. Our results are not so different from the ones reported by Zahid et al. [2] in the same zones of the city. It is interesting to note that the presence of FLAs in domestic and recreational waters is not limited to underdeveloped countries such as Pakistan; recent studies have indicated the presence of such pathogenic amoeba in chlorinated tap and drinking water distribution tanks in developed countries such as China and Australia [33, 34]. In our study, the other sample with the indication of *Naegleria* spp. was from the Lahore canal that is used for irrigation purposes of the land outside the city and another from a private swimming pool. Since the genus *Naegleria* is amphizoic that can survive in water as well as soil, the presence of its species in the muddy water of the canal would not be surprising. For this reason, a study by Zain et al. [35] recommended that people should be discouraged from using the local canal water. The presence of *Naegleria* spp. in the swimming pool is a serious issue, and the recent case of *N. fowleri* reported in Lahore corroborates our result. It was of a man who had the history of swimming in a local pool although the source of the fatal infection was not confirmed [35].

In conclusion, we detected trophozoites in DWDS of mosques and homes and Lahore canal water samples with plaque formation. The screening through PCR confirmed the presence of *Naegleria* spp. in the samples from three zones (Data Gunj Bakhsh zone, Samanabad zone, and Iqbal zone), Lahore canal, and private swimming pools. Even though we did not determine the presence of *N. fowleri* in our study, still the presence of *Naegleria* spp. is enough evidence for a possibility of its presence as well as a strong base necessitating the routine screening of DWDS resources meant for everyday use. However, the precise identification of *Naegleria* spp. is strongly recommended in future studies through the analysis of the sequences of the positive amplicons for a more comprehensive analysis. Furthermore, the development of mitigation strategies is recommended to reduce amoebic contamination, including enhanced chlorination, routine system flushing, and regular infrastructure monitoring, along with public awareness campaigns about the risks posed by FLAs.

## 5. Limitation of the Study

Our study focused on the presence of FLAs in DWDS of the nine zones of Lahore. The study was limited by the lack of facilities for the sequence analysis of the positive amplicon of the positive samples for *Naegleria* spp. This analysis could further give an idea of the species prevailing in the system. Additionally, the study could be improved by determining the microbial population in the water samples that are supportive of such FLAs.

## Figures and Tables

**Table 1:** Epidemiologic and microscopic data of free-living amoebas (FLAs) in water samples from mosques and homes, Lahore.

**Zones**	**Sampling site**	**Temperature (°C)**	**pH**	**Trophozoites detected**	**No. of plaques**
Data Gunj Bakhsh zone	Qurtaba mosque	42	6.9	Y	3
	Ichra mosque	40	6.5	Y	6
	Faiz Alam mosque	37	6.8	Y	3
	Australia Chowk mosque	39	6.5	Y	1
	Kareem Park mosque	32	6.3	Y	8
	Rajgarh home	30	6.9	N	0
	Muzang home	39	6.7	Y	9
	Upper mall home	37	6.7	Y	3
	Outfall road home	38	7.1	N	2
	Kareem Park home	32	6.3	Y	5

Samanabad zone	Sodiwal mosque	36	6.9	Y	8
	Samnabad mosque	42	6.1	Y	1
	Pakki Thatti mosque	30	7.2	Y	3
	Yateem Khana mosque	34	6.9	Y	1
	Gulshan-e-Ravi mosque	27	6.7	N	0
	Sodiwal home	36	6.8	Y	4
	Samnabad home	40	5.9	N	0
	Pakki Thatti home	29	6.7	Y	5
	Yateem Khana home	34	6.5	Y	1
	Gulshan-e-Ravi home	27	7.2	Y	1

Ravi zone	Saggian Pul mosque	39	6.7	Y	1
	Dehli Gate mosque	36	6.9	N	0
	Shahdra mosque	34	6.9	Y	2
	Badami Bagh mosque	33	7.2	Y	11
	Begum Kot mosque	42	6.8	Y	2
	Badami Bagh home	44	7.1	Y	3
	Begum Kot home	45	6.9	Y	2
	Shahdra home	37	6.9	Y	1
	Dehli Gate	36	6.5	N	0
	Shah Alam home	33	7.1	Y	0

Shalamar zone	Chah Meeran mosque	37	6.9	Y	3
	Baghbanpura mosque	32	6.4	Y	0
	Peoples Colony mosque	31	6.3	Y	2
	Faiz Bagh mosque	29	6.4	Y	2
	Mint stop mosque	28	6.2	Y	2
	Chah Meeran home	37	6.9	Y	8
	Mint stop home	36	7.0	Y	1
	Faiz Bagh home	34	7.2	N	0
	Shalamar home	43	6.9	Y	3
	Baghbanpura home	30	6.6	Y	0

Wagha zone	Salamat Pura mosque	33	6.1	Y	1
	Hadyara mosque	29	6.5	Y	4
	Barki mosque	30	6.4	Y	1
	Lakho Der mosque	31	6.3	Y	1
	Daroghawala mosque	33	7.2	Y	1
	Salamat Pura home	33	6.1	Y	0
	Barki home	29	5.9	Y	1
	Lakhoder home	33	6.7	Y	1
	Sultan Mehmood home	31	6.8	Y	1
	Daroghawala home	35	6.9	Y	1

Gulberg zone	Fazil mosque	38	6.9	N	2
	Bibi Pak Daman mosque	25	6.8	Y	1
	Railway colony mosque	33	5.9	Y	1
	Peer colony mosque	36	6.9	Y	1
	Dars Bara Mian mosque	27	6.8	N	0
	Gymkhana home	36	6.8	Y	5
	Society home	38	6.7	N	0
	Peer colony home	36	6.9	Y	1
	Dars Bara Mian home	31	6.7	N	0
	Naseer Abad home	32	6.8	Y	1

Nishtar zone	Basti Saydan mosque	33	6.6	Y	1
	Gajjumata mosque	32	6.6	Y	1
	Kahna mosque	29	6.5	Y	2
	Green town mosque	33	6.9	Y	1
	Farooq colony mosque	27	7.1	Y	2
	GOR 2 home	39	7.1	Y	0
	Kahna home	29	6.3	Y	1
	Kamahan home	33	6.6	Y	2
	Green town home	33	7.0	N	0
	Farooq colony home	29	6.5	N	0

Iqbal zone	Mansoora mosque	35	5.9	Y	1
	Sabzazar mosque	32	6.5	Y	3
	Khokhar Chowk mosque	29	6.4	N	0
	Dholanwal mosque	34	6.6	Y	2
	Johar Town mosque	35	6.9	N	0
	Dholanwal home	28	6.7	Y	5
	Johar Town home	29	6.6	Y	10
	Khokhar Chowk home	37	6.5	Y	2
	Mansoora home	35	6.8	N	0
	Sabzazar home	37	6.5	Y	2

Aziz Bhatti zone	Fateh Garh mosque	29	6.9	Y	2
	Tajpura mosque	30	6.5	N	0
	Mughalpura mosque	38	6.7	Y	2
	Harbanspura mosque	36	6.9	N	0
	Ghazi Abad mosque	32	6.3	Y	1
	Mughalpura home	32	6.7	Y	1
	Harbanspura home	30	6.6	Y	1
	Fateh Garh home	29	6.9	Y	2
	Tajpura home	30	6.5	Y	0
	Ghazi Abad home	33	6.3	Y	1

*Note:* A partial correlation was run on the data to determine the relationship between temperature and pH while controlling for the presence of trophozoites. There was a positive partial correlation between temperature (33.70°C ± 4.29°C) and pH (6.67 ± 0.317) while controlling for the presence of trophozoites (0.78 ± 0.41), *r*(87) = 0.150, *N* = 90, whereas zero-order correlations indicated that there was a negative correlation between pH and the presence of trophozoites *r*(88) = −0.108, *N* = 90, indicating that pH had very little influence in controlling for the relationship between temperature and presence of trophozoites.

## Data Availability

The data used to support the findings of this study is included in this article, and the original data can be requested from the corresponding authors.
